# Principles of a Physiology of the Animal Nature, Peculiar to Animal Bodies

**Published:** 1847-07

**Authors:** 


### ART. XII.

1. *Erste Gründe einer Physiologie der eigentlichen thierischen Natur thierischer Körper: entworfen von* Dr. JOHANN AUGUST UNZER.—*Leipzig*, 1771.

*Principles of a Physiology of the Animal Nature, peculiar to Animal Bodies.* By Dr. J. A. UNZER.—*Leipsic*, 1771. 8vo, pp. 734.

2. JOHANN AUGUST UNZER'S *Physiologische Untersuchungen. Auf Veranlassung der Göttingischen, Frankfurter, Leipziger und Hallischer Recensionem seiner Physiologie, &c.*—*Leipzig*, 1773.

UNZER'S *Physiological Inquiries: in reference to the different Reviews of his Work on the Physiology of the Animal Nature.*—*Leipsic*, 1773. 12mo, pp. 149.

THE Principles of a Physiology of the Animal Nature peculiar to Animal Bodies, propounded by Unzer, is a work on Vital Dynamics generally, but specially on the Dynamics of the Nervous System.

In our original prospectus, issued twelve years since, we announced our intention of giving *retrospective reviews*: our readers, therefore, ought not to be altogether unprepared for the present article. But in truth it was not to fulfil our original intentions that we undertook the examination of Unzer's book, but rather that having our attention called to the volume during our investigation of the doctrines of Prochaska, we were struck by the lucid views and close reasoning of the author; and indeed, as to some points, by the novelty of the opinions propounded. We learnt too, that while anatomy had advanced so largely, vital dynamics had made little progress; or if it might be allowed that in some points of doctrine the science had advanced, in others it had really retrograded. In short, we felt that on the whole the movement onwards of the philosophy of life was "*cum exili et quasi contemnendo progressu,*" and that good service would be done to science by reproducing doctrines like those of Unzer, and giving them that wider scope of application which an improved anatomy admits of, and which, indeed, the loud claims to a newer and truer philosophy of certain modern neurologists imperatively demands. Nor is the review of such a work less interesting and amusing than it is instructive. We witness a powerful mind extending its *palpi* into paths which we now traverse freely, and trying to penetrate a gloom which to us is effulgent light. We anxiously watch his approach to some truth now familiar to us; and then see his steps turned away just when about to seize it and emerge from the surrounding darkness. But we see, too, that if he have turned away from truths lying at his feet, he has overlooked them because he has been engaged in straining his eyes towards the far horizon; and that it was the wide extent of his view which led him to overlook the near and the obvious;—the grandeur of his conceptions, which wiled him from the full enunciation of those principles on which others have since hoped to establish an exclusive reputation.

In introducing the subject of his book to the reader, Unzer sets out by placing an imaginary corpse before himself. Regarding this attentively, he sees nothing there but matter subject to the ordinary forces of matter. There are solids with the property of cohesion; fluids subject to the laws of hydrostatics. The arteries are tubes contracting on the finger in virtue of their physical elasticity, and receiving the injections of the anatomist simply according to the laws of hydraulics. The heart beats not; the contracted muscles maintain the limbs in a motionless position; the machinery of the body is still.

In the living organism matters are far otherwise. It is true the elementary forces of matter act on its components, but other forces are seen in operation upon them, acting according to other laws, and in a definite order. The stimulus which excites no motion in the lifeless heart, in the dead muscle, or in the devitalized arteries, maintains the circulation in the living body, alters the pulse, and excites contractions of the muscles and movements of the limbs. These forces which the living animal possesses, in addition to those of the dead, are "the proper animal forces or powers," (die eigentlichen thierischen Kräfte,) and give it that nature which the author terms "the proper animal nature of animal bodies," (die eigentliche thierische Natur thierischen Körper.)

The reader will better understand the analysis of Unzer's doctrines if he will form a distinct and definite conception of what the author means by the word *Kraft.* This word corresponds to the Latin *vis,* and to the English words force and power. The *force* of gravity is the *power* of attraction inherent in matter; the centrifugal *force* is the *power* of repulsion. The two words are continually used as synonymous, but they are not strictly synonymous. Force is power in the abstract; power is force in action, according to law and arrangement. The elastic force of vapour is the power of the steam-engine. The term power has been used also to express the property or quality of a body; as, for example, the "conducting power" of various substances has been ascertained by experiments in thermotics and electricity; how incorrectly, if the two words, force and power, be synonymous, is made manifest by simply substituting the word force for power, for we have then the conducting force of iron as regards caloric, the conducting force of water as regards the magnetic imponderable. That Unzer means to use the word *Kräfte* to signify primary powers or forces of living matter, just as we speak of the force of gravity, the power of attraction, &c., is manifest from the context and the sentiments of the whole work:

"The ordinary science of physiology considers the forces or powers (Kräfte) of animal bodies in their natural condition and as they act together in connexion with each other, but without distinguishing the simply physical and mechanical from the proper animal forces or powers (Kräfte). This presupposes that we know the laws according to which each of these separate forces or powers (Kräfte) operate separately, and indeed as to the physical and mechanical, whose laws are known, there is generally in reality no difficulty. The physiological works of Haller teach us, in a manner almost impossible to be surpassed, the mechanism of all parts of the animal body, of which the structure develops the functions according to the laws of mechanics, hydrostatics, hydraulics, optics, acoustics, &c. But do we know those laws by which the proper animal forces or powers (Kräfte) govern the body when acting separately from the physical and mechanical, and independently of them? Truly no! or at least very imperfectly. The thoughts and desires or emotions of the soul or mind [Gedanken und Begierden der Seele] are animal moving forces or powers (Kräfte) of the animal body. But do we at this moment know anything of the laws by which these forces regulate their appropriate mechanism (Maschinen)? or have we hitherto troubled ourselves to observe the operation of these laws in each particular class of ideas and desires? We have disputed stoutly enough, whether the soul be matter or brain; whether thought be an electric fire or a movement of the vital spirits; whether souls and bodies exercise a real or an ideal influence on each other; whether souls form their bodies, or whether they are diffused through them or dwell only in the head; whether an instinctive impulse (Trieb) or a passion belongs to the body or the soul; or whether the vital spirits be elastic or inflexible, electrical or etherial, &c.; and all this has been done without any beneficial results whatever, but rather the contrary." (Preface.)

It appears, then, that Unzer had in view a clear and definite object, and that was to rival Newton and the physical philosophers (the physiciens, as they are yet termed in France), by demonstrating the existence of a new and independent class of forces or powers inherent in living matter, and by definitely fixing the laws by which they act. To facilitate the comprehension of his views, we will designate these forces or powers as the *vital*, —or, if that term be too hackneyed, as the *biotic*—forces or powers.

Seeing how much philosophers had erred in their inquiries, on the one hand, by referring, with Stahl, all the animal forces or powers to the mental force only; or with the mathematical and mechanical, by excluding the idea of vital forces, and referring everything to the laws of matter only, our author was induced to reconsider the question, to separate the vital forces from the physical, and to analyse and arrange the former according to their manifest phenomena; in short, to consider *the physiology of the proper or peculiar animal* (*biotic*) *nature of animal bodies*, as distinct from the physiology of the whole animal mechanism. He acknowledges the imperfect nature of his views, but modestly expresses a hope "that some other and better students of animal nature, of which our time affords so many, and with whom he is not to be put in comparison, stimulated by these researches, may draw up and produce a much more comprehensive system," he being contented with the honour of having struck out the idea; or, if he might flatter himself that some much more eminent person than himself, adopting his plan of a physiology, would bring out a pathology of proper biotic nature, he would esteem himself fortunate in having the honour of paving the way to such an improvement in the art of medicine.

*Plan of the work.* Unzer having explained his general views and objects, gives a sketch of the plan he proposes to adopt in developing the laws of the biotic forces or powers. Their seat or origin is in the so-called proper animal machinery or mechanism, namely, the brain and nerves, furnished with the vital or animal spirits, or nervous fluid (Lebensgeister—Nervensaft), through which they are communicated to the mechanism or machines of the body. These "machines" of the body are in fact the *organs* of the body as displayed by anatomists, and are divided into two kinds, according to their uses. Those are simply mechanical in which the laws of physical science are seen operating, as the bones, considered as levers; the tendons as pulleys, the arteries as tubes, &c.; while those are "*animal*" which are directly subservient to thought, and in which the forces of life are seen to act. The term *maschinen* is evidently used as correlative with the biotic forces or powers: these put in motion the "*machines*" just as the elastic force of vapour puts in motion the steam-engine. As, however, Unzer considers muscular fibre "a machine," the term *organ* is scarcely synonymous with "*maschinen*," we shall therefore translate it literally,—machines, thinking it better to sacrifice elegance to a truthful expression of the author's meaning. The animal or biotic machines are the brain and nerves, or the whole nervous system; the mechanical machines are the muscles, tendons, skin and membranes, blood-vessels, glands, viscera, &c. Many of these latter are, however, considered to be endowed with biotic forces, but only secondarily, by means of the nerves ramifying through them. This will be referred to again.

Biotic forces may act either with or without the consciousness and will of the animal—or, literally, the thinking force or power—*Vorstellungskraft*. Now, this is a new force, and it will be well to ascertain the precise meaning our author wishes to express by it, since it plays an important part in his physiology. Looking into the context we find it is used in two or three senses. It means soul or mind, since it is used synonymously with "Seele." It further means sensation, or consciousness of an impression, or of the changes induced thereby in the brain, so that an animal with this kind of "Seele" (*unvernüftige*) is a sensational animal as opposed to an animal with reason—*vernüftige Seele*. It is also used to signify the power of thought and will, or mind, in its highest meaning, and in this sense the *Vorstellungskraft* can excite or originate those material changes in the brain, which are necessary to thought and volition, as well as be conscious of them when excited by impressions only. Thus sensation, or consciousness of impressions, is not, in Unzer's views, a primary force, since it cannot act on the brain or system generally without being excited; on the other hand, the mind or soul is a primary force, inasmuch as it can originate material changes in the brain and nervous system.

There are a few other terms, whose precise signification it will be subsequently necessary to define, but the preceding is sufficient to enable us to understand the author's plan.

The First Part considers the physiology of the brain, and includes the operations of the mind, whether sensational or reasoning. In the Second Part the physiology of the spinal cord and nerves is expounded, and *reflex phenomena* are fully defined and analysed. In the Third Part we have the economy of the *whole* of the *animal* forces or powers considered; a definition of animated nature and an arrangement according to leading types, and especially indicating animals without consciousness; animals with sensation only; and animals with reason. And lastly, an essay on the origin, life, perfection, decline, and death of animal nature. Such is the plan of Unzer's work as laid down by himself; and, after again modestly acknowledging imperfections, inviting his readers to do better than he has done or can do, declaring his willingness to profit by fair criticism, and his determination not to notice personalities, he addresses himself to his task.

In the first chapter of the first part, he states his views of the nature of the machines subservient to the biotic and psychical powers of animals, or in other words, of the anatomy of the nervous system in man and the lower creatures; these are principally taken from Haller. The brain—the seat of mind, or "the soul"—and the nerves, are the two great portions of the nervous system, the latter all ending in the former as the great centre; the spinal cord being considered according to the current doctrines, as in some degree a bundle of nerves. With regard to the ganglia, he thinks that, like the brain, they are endowed with the property of reflecting impressions.

"It is also a settled fact that the ganglia are always larger than the nerve arising from it, and sometimes very remarkably so. It is also certain that almost all the ganglia give off more nerves than they receive, and that they also as well as the brain give origin to nervous twigs; whence it is very probable that they also, like the brain, reflect certain impressions that they have received, to the limbs they govern along the new nerves that spring from them, although the impressions do not in this case become objects of consciousness, as in the brain. But more of this afterwards." (p. 30.)

The nerves are motor and sensory, and are endowed (in common with the brain) with the vital spirits or nervous fluid, (Lebensgeister—Nervensaft) the office of which is to carry impressions to and from the brain along the nerves. It is formed from the blood-vessels in the gray matter of the brain, and may be increased or diminished, and the functions of the nervous system changed accordingly. Whatever impairs its production in the brain, or hinders its transmission along the nerves, interrupts the action of the powers or forces of the mind and the nerves. Thus congestion, or compression of the brain, or division of the nerve, will stop the operations of the vital spirits or nervous fluid. On the other hand, sleep, wine, and alcoholic stimulants, agreeable passions, pleasant mental stimuli, &c., increase the quantity of the vital spirits and correspondingly exalt the functions of the nervous system. All this, however, is stated by Unzer in deference to authority only—that of Haller. Having propounded the current opinions, he dismisses them with the remark, that little is known respecting the nature of these vital spirits, and that little is very uncertain and at the best only probable; and further, that they really are not necessary to the study of the laws of the proper animal forces. Henceforth therefore, he says no more about them. He seems glad to get rid of them, but influenced by conventional feelings, buries them in forgetfulness with ceremonial respect. In his answer to one of his reviewers, he again maintains that the theory of "vital spirits" is not necessary to his views.

*Physiology of the brain :* its biotic forces considered as bio-psychical forces (thierische Seelenkräfte). After observing that the alternate elevation and depression of the brain is a mere mechanical movement, and dependent on physical forces, Unzer proceeds with the proper physiology of the brain as a vital or biotic "machine." First, he observes that the brain is the seat of mind or "the soul,"—that is a fundamental proposition which all experience proves, conflicting statements to the contrary notwithstanding. It is also a compound organ, for certain parts being injured certain conceptions or sensations do not occur, and the ideas, desires, and instincts connected with them are prevented or disappear. The property or power which Unzer attributes to the brain in particular, is that in virtue of which those changes that are necessary to every act of thought are excited; but he shall speak for himself.

"When a thought arises in the mind, a change must necessarily occur concurrently therewith, without which the conceptive faculty (Vorstellungskraft) cannot act; and when this change occurs in the brain, the conceptive faculty is necessarily excited into action. Whatever may be reasoned on the matter, a change in the brain must consist in a movement (Bewegung), and the medullary matter must also be endowed with a power of exciting movement (Bewegendekraft), which acts in harmony with the mind (Vorstellungskraft). So that each distinct class of perceptions is always connected with a certain animal (biotic)

movement, and with these movements a certain class of perceptions, for it is ascertained from numerous observations that after certain injuries of the medullary portion of the brain, especially that part from which nerves of sensation arise, certain kinds of perceptions, as for example, certain sensations (Empfindungen) are prevented or disappear, and together with them all the ideas, desires and instincts dependent thereon, as well as other faculties of the mind. This power of the brain to excite movement (die Bewegendekraft) which is thus connected with the mind and power of thought is an animal soul force (thierische Seelenkraft), and hence arises *the fundamental general principle in the doctrines of the connexion between body and mind, that the medullary matter of the brain possesses an animal soul-force* (thierische Seelenkraft), *by means of which at every act of mind* (Vorstellung in der Seele), *whether it be a sensation* (Empfindung), *imagination* (Einbildung), *desire* (Begierde), reflection (Betrachtung), *or influence* (Einfluss), *it* [the brain] *produces in itself a certain animal movement necessary thereto, without which the act of thought can neither arise nor continue, and with which it infallibly arises and continues.* This animal soul-force is peculiar to the brain, and is the property of no other animal machines, because in none other except in the brain does an animal movement develop perceptions. The medullary matter of the brain can also with propriety be designated the only instrument of the conceptive power of the mind, by means of the animal movements of which it [the mind] puts its force into action, and continues it [in action] and without which [the animal movements of the medullary matter] it would absolutely remain inactive. Philosophers have already introduced the phrase *material ideas* to express those movements in the brain that are necessarily connected with each act of thought. (Baumgärtner's Metaphysics, § 416.) A psychological materialist considers these material ideas as the ideas of the mind itself. But since it must be firmly maintained that the thinking faculty is an immaterial substance (Substanz), it cannot certainly be granted that these changes in the brain are really ideas; but since the two are inseparably connected with each other, and the mind never acts without these movements nor can act in animals, it is fully established that every act of thought presupposes and causes a movement in the brain (material idea), and every such movement in the brain presupposes and causes an idea in the mind (Vorstellung in der Seele.)" (pp, 41-2.)

Unzer's doctrine of "material ideas" differs in little respect, if any, from that of the most esteemed metaphysicians. All acknowledge and maintain that a change takes place in the brain with each act of thought. Reid, for example, says, "In perception the object produces some change in the organ; the organ produces some change in the nerve; and the nerve produces some change in the brain." Newton, Dr. S. Clarke, Locke, Malebranche, Hooke, Descartes, Bonnet, and others held similar doctrines. In adopting it Unzer expressly cautions his readers in a special note against a misapprehension of his meaning. He has no intention of giving the notion that there are hieroglyphical figures on the brain, or impressions literally made upon it. Unzer's "material ideas" are altogether different from Dr. Laycock's "psychical substrata." Dr. Laycock wishes to express by that term, certain changes in the molecular constitution or arrangement of the neurine induced by frequently recurring *impressions communicated from without,* and corresponding to those impressions and to none other. These "psychical substrata" are therefore part of the constitution of the body; consequently like other organic properties, are transmitted from parents to offspring, and constitute the foundation of characteristic or acquired instincts, propensities, &c.*

Unzer uses as synonymous with "material ideas" the term "impressions,"

* Essay on the Reflex Function of the Brain.—By T. Laycock, M.D.—British and Foreign Medical Review, Vol. XIX, p. 308.

inasmuch as by the latter he wishes to signify the continuous changes which continued operations of the mind excite in the brain. He also uses, but once or twice only, the term Bilder der Vorstellungen—images of conceptions, to express the same thing. However, he expressly maintains that the strength of a perception or conception is in proportion to the activity of the movement, or in other words, to the intensity of the material idea. A large material idea comprises or is made up of smaller ones, and this constitutes a large conception (grössere Vorstellung). For the term "animal soul-force," used to designate the efficient cause of these changes, and which is peculiar to the brain (quotation, p. 186), we shall substitute the term cerebral power.

Conceptions may be of two kinds. The one are dependent on movements in the brain (material ideas), which are induced by impressions on the senses; the other are dependent on movements of the brain (material ideas), induced by the conceiving force or power of the mind. The former are termed sentient or sensational (sinnliche) conceptions, or simply natural conceptions; the latter are termed arbitrary, or spontaneous, or "physiologically free." These terms are derived from Baumgärtner, who appears to have followed Locke. Unzer specially distinguishes between these two classes of ideas or conceptions. Those of sensations are altogether dependent on impressions made on the brain; the ideas of the intellect are dependent on the immaterial essence—the soul. As no conception can occur without this change in the brain termed a material idea, Unzer proceeds to inquire how these changes are excited in the brain, and what actions they induce in the animal economy; and this leads him to the consideration of the biotic force of the brain, for that is the force which develops the material ideas, and his first chapter is on external sensual impressions.

*The biotic properties of the nerves.* The nerves, Unzer observes, have two points; the one is that connected with the brain, the other is the termination in the body, or on the surface. If either end be irritated, the irritation is propagated to the other extremity. If a nerve be irritated in any part of its course, irritation is propagated in both directions; and if it be cut, and the cut ends irritated, if that next the brain be touched, the irritation is propagated to the brain; if that next the periphery, the irritation goes to the periphery. When an animal apparatus (a nerve) is so influenced, either by a touch or a transmitted movement, or by any other impression of whatever kind it may be, that animal actions (Wirkungen) are excited, the change thus induced in the "machine" is termed a sentient impression (sinnliche Eindrück) or nerve-feeling (Nervengefühl). Thus, a sentient impression made on a nerve will travel from the cerebral to the peripheral extremity, and *vice versâ.* If it be made on the peripheral extremity or on the trunk of the nerve, and goes on to the brain, Unzer distinguishes it as an *external* sentient impression: or as a synonym, a "nerve-feeling *from without inwards.*" But the irritation must excite changes in the animal "machine" to become an impression. Light may be applied to the most delicate and most exposed nerves of the skin, but it excites ordinarily no external sentient impression. The excitation of the requisite change is purely animal, and quite unconnected with the mechanical or physical laws of matter. When the medulla of the nerve is touched, and the most energetic animal actions follow, as conceptions of the mind; or movements are excited by the cerebral forces, or simply by the biotic forces, not the slightest change is observable in the nerve itself. Very often indeed it happens that the physical force of the impression has no relation to the amount of action excited, for the slightest touch is often followed by greater results than a strong pressure, thrust, &c. Nor is there anything in the mechanical structure of the nerve itself which can explain the propagation of the change or impression.

It is an unfortunate circumstance that we have no English word which expresses this faculty or property of living matter to receive and react on impressions, and which is as much a property of the microscopic cell, as of the nerve of special sense with its highly organized apparatus. By Unzer it is termed their Sinnlichkeit, which may be translated sentientness, or sentiency, and the change itself, which the nervous matter undergoes on the application of a suitable stimulus, a *sinnliche Eindruck,* which we have translated as *sentient impression.* The phenomena resulting from that change would therefore be properly characterized as *sentient* phenomena.

The great objection to this and all other expressions derived from the Latin verb *sentio* is this, that, let the true meaning be guarded by the context ever so strictly, as not implying consciousness or any mental act, still they are continually considered to imply the latter, because in ordinary language these terms invariably do imply consciousness. A sentient being is a percipient or thinking being. Whytt attributed these phenomena to a "sentient principle," as opposed to a "rational principle," but the use of this phrase, although expressly defined by him as not implying consciousness, led to a complete misapprehension of his views, as we have formerly shown (Vol. V, p. 524). *Sensation* and *sensibility* have also been very commonly used by numerous writers to express the cause of these phenomena, as by Prochaska (Vol. XXIII, p. 212), Alison, and many French and German physiologists of the present day. These terms have, however, given rise to the same misunderstanding as the preceding.

Some writers have designated these phenomena from the mode in which they presumed them to be produced. They have thus been termed sympathetic actions, involuntary, excited, excito-motory, reflex acts. Several objections have been necessarily raised to such terms. In the first place, they do not sufficiently mark one of the leading characteristics of the phenomena, namely, their adaptiveness to the well-being of the organism. Secondly, the vital mechanism in which they take place is incorrectly limited either to a "sympathetic system," or a "true-spinal system," &c. Thirdly, the sentient-like characteristics of the phenomena are pressed as strongly opposed to the more material causation advocated by this class of physiologists; thus, the Stahlians referred them to the operation of the unconscious *anima;* Whytt (influenced, doubtless, by the Stahlian doctrines) to an unconscious sentient principle; and others to sensation or sensibility.

It is certainly impossible to leave the leading or chief characteristic of adaptiveness out of consideration, in devising a name for the class of phenomena referred to. Adaptiveness is seen throughout the whole of creation—in the planetary system as well as in the terrestrial organisms. The vital changes and actions resulting in organized matter from suitable stimuli are *like* those of sentient beings, but it is plain they are not the result of individual thought or mind. On the contrary, they are manifestly the result of a power of adaptiveness in matter, acting universally, and therefore in all organisms without exception, and subject, like the universal property of gravity, to general laws. Of this power we can know nothing but by its effects; we can only say, they are *like* those of sentient beings, and the property is sentient-like in its operations. We will therefore venture to translate this into an unappropriated Greek term, namely, homæsthetic, from *ὅμοιος, similis,* like to, and *αἰσθητικὸς, sensu prœditus,* sentient.

The property of living matter, then, whereby adaptive actions and changes are excited in that matter, independently of volition or consciousness or thought, is homeoæsthesis, or abbreviately, homæsthesis, and the actions and changes themselves are homæsthetic, and this without reference to the class of organisms in which they occur, that is, whether they be animal or vegetable.

To recapitulate. When by rays of light, a change in temperature, or the contact of matter in any form, a vital change is induced in a nerve or ganglion,—that is an impression; if mere adapted actions result without consciousness, the impression, actions, and changes are homæsthetic; if there be consciousness of any of these, it is a sensational impression, and the actions are psychical. That the reader may the more easily comprehend Unzer's views, we shall substitute these terms for his own polysyllabic and uncouth phrases: for example, the phrase *sinnliche eindrücke* will be properly rendered by the term *impressions,* simply, as that includes the idea of adapted actions expressed by the word *sinnliche.* We allow that as few new words should be introduced into science as possible, but if any of our readers will improve on our coinage, they will do the literary state some service.

When a nerve of an animal possessed of mind receives an external impression, it is either propagated onwards to the brain, or is arrested in its course thereto. If it arrives at the brain it there excites through the nerve "material ideas," and their corresponding conceptions or perceptions. These latter are termed, when thus excited, external sensations (aüssere Empfindungen), and the power of the nerves to excite them is termed the sensational power (empfindende Kraft), or simply *sensibility* (Empfindlichkeit).

Unzer explains that the word Empfindung has three meanings. First it designates the involuntary perceptions to which impressions made through the senses give rise; these are external sensations (äussere Empfindungen). Secondly, the consciousness which the soul has of its own existence (Bewustseyn); this class of perceptions he designates internal sensations (innerliche Empfindungen). Thirdly, the verb Empfinden—to feel, is used indefinitely to express the perception of the existing condition, whether excited by an external impression or not. Impressions, then, *with* consciousness are sensations, or sensational impressions; those belong to the psychical actions. Impressions *without* consciousness, are *homæsthetic* impressions, or simply impressions. The nerves appropriate to the unconscious or reflex acts are *homæsthetic;* those connected with the mental acts are *sensible.*

Unzer next notices certain phenomena connected with the nerves, as for example, the power which we possess of referring sensations to the point of contact of the impression, and the circumstances that when a nerve is touched in any part of its course, we refer the point of sensation to the extremity of the affected nerve. He illustrates this by the sensations experienced as if in amputated limbs. He also maintains that the nervous twigs are isolated throughout their whole course, and that each have a point in the brain in which the external impressions they convey to it develop "material ideas." He notices the difference in our sensations, as of warmth, cold, hard, soft, dry, moist, &c., and the differences in painful sensations, as, for example, a blow so severe as to fracture a limb, or a slashing of the flesh gives less pain than a caustic or burning application; but he sees no means by which these differences can be explained.

The circumstances that determine the transmission or non-transmission of external impressions to the mind are specially noticed by Unzer; and he deduces from them the general functions of the brain and nerves, and sympathetic ganglia. He observes specially, that many external impressions are made on the nerves which excite movements, but which never arrive at the mind. Thus, the mind does not perceive the sensation of things sweet, bitter, &c. received into the stomach, but the result of anything sweet or bitter which is irritating, is to excite vomiting. So the blood streaming into the heart, excites as the originator of an external impression on the nerves of the heart, the movement proper to that organ, but the mind *perceives* no impression. That the heart's action is independent of the mind is proved by the fact, that its movements may be excited when removed from the body. The seat of these motive powers he places in the ganglia, and he looks upon these as natural hinderances or obstacles to the transmission of impressions to the brain. Unzer also reviews other causes, which will prevent the production of external sensation. First, continuity of the nerve is necessary; then continuity of transmission; then a proper condition of the brain for the reception of the impression and the formation of material ideas. One hinderance to these is sleep, and another the habitual reception of external sensations. He enumerates five modes in which the habit of sensations render their resulting action weaker. On the other hand, the external impressions may be transmitted to the mind with greater facility, or may induce more powerful results, or the contrary; and this condition is termed being sensible, or insensible, and is the foundation of the doctrine of idiosyncrasies and temperaments.

After a short notice of the senses, Unzer proceeds to consider the sensational conceptions (sinnliche Vorstellungen.) No animal thinks without sensation (ohne zu empfinden.) But the conceptions of the mind may be of various kinds. They may be dependent on the material ideas excited in the brain by external impressions, or on material ideas excited by the mind, independently of external impressions. He adds, however, that upon a more careful consideration it will be found that the most "arbitrary" or "spontaneous conceptions" are dependent upon external impressions; but the connexion is so remote in some, and so immediate in others, that a discrimination is necessary. When spontaneous conceptions arise (which are none other than imperfect external sensations,) in so far as they belong to former external sensations, they are Einbildungen, imaginations, ideas of memory, and in so far as they belong to the future are Vorhersehungen, foreseeings—anticipations.

Unzer proceeds to illustrate these and other views (which we omit to notice), as to the nature and formation of simple notions or conceptions, and abstract ideas by a reference to examples. Thus, dreams are the Einbildungen—the sensational conceptions of the sleeping person. In sleep-walking, and in insanity, these become so vivid as not to differ from those excited and developed naturally. This view of the identity of the psychical states of dreaming and insanity, was stated to be the principal object of M. Moreau's work, in our review of the book (vide p. 220 of this Volume). When a conception is reiterated or renewed, and the mind recognises it as one that has occurred before, that is memory.

Unzer thus theorizes on the origin of a set of ideas opposed to those of memory—ideas regarding the future.

"The sensational foreseeings (Vorhersehungen) and expectations (Erwartungen) arise out of existing external sensations, and sensations of memory, each having a part in common with the other. The mind in these considers that as belonging to the future, wherein both are different, or *foresees;* and when it considers this as the same with that which is felt as actually coming, *it expects* (erwartet). Simply sensational anticipations are forebodings—ahndungen." (p. 85.)

All these conceptions have their corresponding "material ideas" in the brain, and are subject to the general laws regulating simple conceptions and their material ideas. They are often developed in dreams and in insanity. *Wahrsager*, or Seers, are persons who have the faculty of anticipating or expecting the future. The understanding, acts of attention and of abstraction, &c., are all explained by Unzer in accordance with the preceding doctrines.

*Nature of desire and aversion.* Passing from the faculties of the mind, Unzer takes up the question of pleasure and pain. He first observes, that we are conscious of a difference in our conceptions, sensations, &c.: many we designate as pleasurable, many as painful. These he argues are essentially different in their action on the brain. If at any time a painful conception becomes pleasurable by being placed in relation with another conception, it becomes by that act a new conception. He further maintains, that there is the same difference in our perception of external sensations, and that they have a different action on the brain according as they are pleasurable or painful. When the mind foresees (vorhersieht) a pleasant thing, or what is the same thing, anticipates the conception of a thing which is pleasing, it exerts its power—it strains itself—to produce or obtain the subject of this anticipated agreeable conception, or to make it present, or to exist; that is, to experience it or feel it (empfinden) in the third sense or meaning of the word (vide ante, p. 188), or to make the anticipation a reality, or if the anticipation be displeasing to produce the contrary condition. In the one case, the condition or effort of the conceiving force is DESIRE, in the other AVERSION. The satisfaction of these impulses, propensities, or anticipations, requires the development of external sensations excited by external impressions. Since pleasure and pain are the moving causes of the efforts, they are consequently the grounds of the desires and aversions; thus considered, they may be termed the exciting motives, momenta—springs—(Triebfedern) of the mind (Gemüths). The impressions of pleasure or pain excite an anticipating sensation, and in so far an effort of the cerebral power sufficiently powerful to induce the material ideas of this future sensation, and this is the "material expression of the desires and aversions in the brain." He who desires, foresees a future pleasant thing or conception, and endeavours to realize it so that it may be actually felt. In each desire three things are to be distinguished: 1. A foreseeing and expecting of a future sensation. 2. The exciting motives of the mind, delight and pleasure; these communicate the impression of pleasure to the "imperfect material sensation" in the brain, excited by the preceding. 3. The spontaneous effort of the mind to realize the entire anticipated sensation; this is connected with the exertion of the cerebral powers to complete the "imperfect material sensation" which the soul foresees. The action of the desires on the animal economy is in proportion to the strength of these phenomena. Aversion is entirely analogous to the preceding, except that the efforts (in the third proposition) are directed to attain the converse of the anticipated unpleasantness. The conceptions which excite pleasure and pain may be either sensational or intellectual; that is to say, may be distinctly connected with the sensations or with the understanding. The former, which excite desires and aversions, are to be carefully distinguished from mere impressions, or impressions without consciousness—the intellectual excite "desires or aversions of the will."

*Nature of instinct and its modifications.* A strong and strictly sensational desire which arises from obscure sensational stimuli, and the material ideas of which are little developed in the brain, is termed a 'blind impulse,' or synonymously 'instinct,' 'sympathy,' 'sensual propensity,' 'sensual inclination,' generally 'natural instinct,' and an abhorrence arising in like manner, is termed an 'aversion,' 'antipathy,' 'sensual disinclination,' 'enmity.' Both classes of instincts are termed animal, and divided into self-preservation, self-defence, the continuance of the species, and love of offspring. Stronger desires and aversions arising out of complex sensational stimuli, of which the person is conscious, and of which the material ideas are much more developed in the brain than those of the sensational instincts, are termed passions, affections, emotions. Both these are subject to the same laws as the simple desires and aversions, and are divided in their operation into the same three stages. The conceptions (Vorstellungen) which are required for the excitement of the desires and aversions, when they excite the conceiving force, or acts of mind, are either sensational or intellectual. The former belong to the instincts and passions; the latter are termed, motives to action (Bewegungsgründe), and excite "desires or aversions of the will." These psychical desires and aversions are subject to the same laws, and act in like manner as those of the instincts. The material ideas of the anticipations and expectations they excite, arouse the cerebral power to efforts directed to the object of realizing the perfect material ideas of the anticipations, or counteracting the aversions. We pass over the remaining purely metaphysical portion of this part.

*Action of material ideas on the brain.* Reference has been continually made to material ideas. Unzer next proposes to consider the operations of these in the proper animal or biotic machinery, the brain and nerves. All occur either through the one or the other, or they set simply mechanical, as opposed to biotic, machinery into motion. Unzer confines the consideration of them to the two classes which act through the brain and nerves. All material ideas are movements (Bewegungen) in the brain, consequently their action can be no other than to excite movements. They are biotic forces. It is the function of the cerebral power to produce material ideas for the conceptions of the mind. Farther, the immediate results of the operation of the material ideas that act on the brain only and do not extend their influence to the nerves, or to the mechanical machines, are simply other material ideas, which give rise to other conceptions, and consequently are developed at other points of the brain distinct from the first. Thus complex ideas are produced.

*Action of material ideas on the nerves.* These material ideas are next considered in their action through the nerves. And first, the impression they make on the brain is carefully distinguished from the impression from without, made by and through the nerves. To distinguish the former class of impressions from the latter, he terms them, inner sentient impressions, "innere sinnliche Eindrücke," while the other are external or outer, as already explained. We shall omit the term sinnliche, in characterizing the inner as well as the outer impressions. The inner impressions are propagated from within outwards, while those received by the nerves are propagated in a contrary direction.

*Two sets of fibrils in each nerve, and their function.* In prosecuting the consideration of this part of the subject, Unzer takes occasion to show that each nerve must run an isolated course from the periphery to its termination in the brain, and vice versâ, and that the impressions of material ideas, or inner impressions, must act upon the point of the nerve at its junction with the brain. This transmission of impressions in the two directions is, he argues, to be attributed to two sets of fibrils in each nerve, just as there are two sets of blood-vessels in connexion with the central organ of the circulation. It is remarkable that his Göttingen reviewer (Haller?) notices this doctrine of the existence of two kinds of nerves in the same trunk, to oppose it. "Further on Unzer considers it probable that there is a difference between the nerves proceeding *to* the brain and *from* it, and thinks the arguments to the contrary invalid. He forgets that the proof rests with himself, and that neither experiments nor anatomy favour his hypothesis." To this Unzer replies by repeating his doctrine of two classes of fibrils in one trunk, and observes, that it is not necessary to his views; nevertheless, certain phenomena can only be explained by the hypothesis; and, therefore, although he cannot prove it to be a fact, it may still be useful as a hypothesis. In fact, his fundamental doctrine is, that there is a reflexion of the impression from the cerebral end of the sensory nerve to the cerebral end of the motor, occurring in the brain itself. (Answer to Rev., p. 25.) Unzer also reviews properties of the nerves now well known and thought new, as for example, the reception of impressions made along the trunk, and the reaction or radiation of inner and outer impressions on the central ends of the nerves; first, in exciting the functions of other nerves, inducing *sympathetic actions*; and, secondly, by frequent repetition, developing the phenomena of *skilled actions,* or what have been usually denominated *habits,* or habitual motions. The influence of attention, or anticipation and expectation is discussed; and it is shown that an influence is sent along the nerves from the brain during this act. He mentions as an example, amongst others, that the papillæ of the tongue in which the nerves are distributed, are excited with the expectation of sugar, salts, &c. The action of attention on the terminal fibrils in the brain may also develop impressions analogous to those made on the periphery of the nerve, and thus imperfect external impressions are formed. These are known only by the abnormal conception excited by them, and which are spectral illusions, false sensations, as of touch, hearing, and smelling, and vertigo. It is impossible not to be struck by the lucid views detailed by Unzer under this head, nor to incline to the opinion that the transference of an influence from the brain outwards is not confined to the motor nerves, but that the sensory or sympathetic constitute a channel of communication in the same direction. If the hand of a nervous female be pointed at she complains of a sensation of coldness; what is this but an "external sentient impression," excited by the action of attention on the cerebral termination of the nerve of touch, with probably a real change in the peripheral termination? That some such influence is sent to the nerves of secretion, as of the salivary and lachrymal glands, kidneys, &c. is certain, and renders the preceding supposition more than probable, and that Unzer's view is the correct one.

*Operations of the mental and cerebral powers on the mechanism or machines of animal bodies.* The *mechanical* machines of animal bodies are, in virtue of their structure, capable of many movements; but they are much oftener excited into action by vital, than by simply physical or mechanical forces. The influence of the soul on the body takes place through the material ideas in the brain, or through the sensational impressions on the brain of their corresponding conceptions; movements of the mechanical machines operated through these media are psychical actions (Seelenwirkungen.) They are effected either mediately through the nerves, or immediately by the material ideas. Now these mechanical machines are the muscles, tendons, membranes, vessels, glands, bones, cartilages, viscera, &c., and not the brain or nerves, which are the proper *animal* machines. The former possess animal forces it is true, but in virtue of the nerves by which they are supplied, and not otherwise.

Unzer then takes a review of the machines of the body, beginning with the brain. The cortical substance, he observes, is not the seat of the psychical forces, nor the instrument of the mind, but only a tissue of blood-vessels and canals—a secreting apparatus—in which the vital spirits are elaborated from the blood; from thence they are communicated to the medullary substance, and through the latter distributed to the whole body; so that the cortical substance is in fact a secreting viscus situate within the cranium. The medullary substance is the seat of the material ideas, but he argues that the latter have a close connexion with the cortical substance, and the secretion from the numerous blood-vessels it contains, inasmuch as so large a quantity of blood is sent to the brain for the maintenance of its functions. Further, that material ideas may have an influence upon the cerebral capillaries, is rendered probable by the fact of their direct and immediate action upon the heart itself and the capillaries distributed elsewhere. It may certainly be stated that mental acts do often induce a disturbance of the circulation within the cranium, but this is inconclusive as to the *direct* action of material ideas situate in the medullary matter upon the capillaries of the cortical substance, because it *may* be induced indirectly through the action of the nerves on the central force-pump, the heart. So that Unzer says the opinion is probable but not certain; nor can it be demonstrated.

With regard to other machines, Unzer observes, that the nerves are in the strictest connexion with them, inasmuch as they form loops at their ultimate termination within the intimate tissues of the machines, as is seen in muscles, and around the blood-vessels. The physiology of muscular action is then set forth, partly by extracts from Haller, and partly in his own words and style. He points out the well-known laws of muscular actions very distinctly, and their relation to the nerves and brain. Material ideas make impressions on the origin of the motor nerves in the brain, and thus excite corresponding muscular actions. If a muscle be immediately excited to action by external impressions, the nerve which receives them excites the action; of course the anatomical distinctness of motor and sensory fibres in the same trunk was not known at the time he wrote, but we have seen that he maintained such distinction as a theory. Perhaps it is also worthy of notice that Unzer considered the biotic powers of the two classes of nerves identical in their nature, and that the difference in their action is dependent upon the difference of the apparatus in which the impression on the nerve finally terminated and excited changes. An impression on a motor nerve or on its trunk is propagated to the machines of movement—namely muscles; an impression on a sensory nerve is transmitted to the seat of the biotic forces and the "machine" of thought, namely, the cerebro-spinal axis; and there develops material ideas and sensations, and is thence reflected as an inner impression on the motor nerve. He adds, action may be transferred to other and far separated roots of nerves, and this is sympathy, and the actions are sympathetic. This kind of communicated action is not, he observes, at all unusual, and is seen when a painful injury that excites spasms in one set of muscles extends its influence to another set. With regard to the action of the material ideas, whether of conceptions of the understanding, or of the instinct, he observes that the muscular action is forcible in proportion to the force of the impressions made on the origin of the motor nerves by the material ideas. The frequent repetition of these impressions determines the form and size of muscles, so that those of the face are at last so formed as to express the thoughts, and a knowledge of their meaning is the science of physiognomy.

The vascular and secreting systems are subject to nervous action, but specially the heart. It has nerves fitted to receive external impressions, to transmit these to the brain, to develop there external sensations or homæsthetic impressions, which, provided no material hinderance occur, are reflected along the same nerves, and thus not only exert a biotic influence, but develop psychical actions. Thus also sensational conceptions, desires, and aversions will act on the heart. Nevertheless, the heart will act even out of the body and separate from it.

*Action of external impressions through the nerves, or reflex acts.* We need not follow our author through his review of the action of material ideas on the other hollow muscles, as the pharynx, œsophagus, intestinal canal, bladder, &c., as it is in all respects analogous to the preceding. We therefore pass on to an interesting chapter of Unzer's work—a chapter, indeed, which may be considered as containing the first elements and original enunciation of the doctrines of reflex action. After pointing out the distinction between acts resulting from impressions only, and those which are true psychical actions derived from the brain, Unzer observes:

"These two really distinct things have always hitherto been considered as immediate psychical actions resulting from external sensations, and thereby the physiological doctrines regarding external sensations have been shockingly confused (greulich wirwirret). It is desirable that we make this important distinction as clearly as possible here, whereby, however, we must presuppose that which in another part of this work *we are the first to demonstrate*, namely, that the external homæsthetic impression on the nerves (the external nerve-feeling) really possesses in itself a biotic moving force before it comes to the brain, to excite these external sensations. The clearest proof that the motion excited in mechanical machines by the touch of the end of a nerve is simply an action resulting from the external homæsthetic impression (feeling), or the external sensation, is to be found in the fact, that the same contact of the nerve which excites the movement may be repeated after its connexion with the brain is broken, or for greater certainty against sympathetic acts, after the whole head is severed from the trunk. The result is, that so long after decapitation, as traces of animal life continue, there are the same movements from a touch of the nerves, although the external impression can no longer be propagated to the brain and excite a material sensation; consequently, it is not the psychical action through an external sensation which this external impression otherwise excites through the brain in the soul, but an animal [biotic] action proper to the peculiar biotic moving force of the external impression. If this experiment respecting the apparent psychical action of external sensations on mechanical machines be investigated, it is found that the biotic motive force of an external impression develops the majority of those movements in the mechanical machines which we have considered to be solely the psychical action of its external sensation, &c." (pp. 179-80.)

Unzer terms these mere mechanical movements dependent upon impressions on the nerves "Nerve-actions," (Nerven-wirkungen, actiones nervorum) to distinguish them from the "soul-actions" (Seelen-wirkungen, actiones animæ). As the majority of the latter in the mechanical apparatus are really the former, he observes that it is wrong to state, that the one cannot be the other. Thus the action of the heart is ordinarily a "nerve-action" or reflex act, for we are not conscious of the external impression which excites that animal apparatus.

If an external impression be not felt, and nerve-actions or reflex acts follow, the action itself may be felt, or become an external impression giving rise to other actions; these Unzer says are also nerve-actions.* It may not be easy, he observes, to say what use sensation is of in nerve-actions, but the result seems to be, that when they are psychical as well as reflex, they are more regular, perfect, and complete. If we were not made sensible of spasm or cramp in the muscles by pain, the "machines" might thereby suffer injury and we not exert ourselves to prevent it, &c.

Looking at the psychical actions in the mechanical apparatus from another point of view, Unzer cautions his reader against confounding the psychical actions resulting from conceptions, and those resulting immediately from external sensations. Thus, if a certain food taken into the stomach has caused us at some past period to vomit, and on seeing or smelling it we vomit again, Unzer argues that it is not the sight or smell which excites the vomiting, but that there exists an idea previously formed (Einbildung), and this being re-excited, acts on the origin of the

* We must request the reader's indulgence for the use of these truly uncouth terms and Germanisms, inasmuch as we cannot substitute others of a more elegant construction without injury to the sense of the author, or without coining new words.

motor nerves, and causes vomiting. Again, if we see a stone falling upon us, we get out of the way; but this is no direct psychical action of the muscles excited by the external sensation of the stone, but a fear of impending danger which the appearance of the stone excited.

*Physiological nature and operation of pleasure and pain.* Unzer next considers the nature and action of pleasure and pain, and lays it down as a fundamental proposition, that all external sensations are either agreeable or disagreeable, and that if in greater strength they cause "Kitzel" and "Schmerz," or *tickling* and *pain.* He thinks that we cannot possibly know in what consists the difference between the impressions causing them; we can only judge by the phenomena. He adds, however, that it appears that that external impression or sensation is agreeable, which is conformable to the natural structure of the nerve, and on the contrary, that that is disagreeable which does not act conformably to the animal structure and force of the nerve, but rather in some degree does violence to it. Further, all movements, of which a mechanical apparatus is capable in virtue of its structure, are either conformable to the natural destination or functions of the apparatus in the condition of health, or they diverge from this conformity. All psychical actions arising from external sensations, stimulate the mechanical machines to which they extend, either to movements conformable to their destination, in which case the external sensations are agreeable, or to the converse, when they are disagreeable. Pain is curative—is the sentinel of the organism,—and excites the movements proper for the removal of its cause; namely, movements opposed to the natural and pleasurable movements. In the hollow muscles, for example, as the gullet, an ordinary external sensation excites the natural propulsive movement, a powerful sensation, or pain, induces a violent morbid movement or spasm. In like manner, pleasing or unpleasing sensations, or injurious or beneficial impressions act upon the vessels, and cause an effusion or efflux of fluid, and so on through the various mechanical machines of the body, as the pharynx, œsophagus, and alimentary canal; the skin and mucous surfaces, (quoting sneezing, excited by irritation of the mucous membrane of the nares, as an example;) the glands and glandular organs; the organs of the senses and the sexual organs. With reference to all the excited phenomena manifested continually in these "machines," Unzer observes, that the law of external sensations which Krüger has laid down in his physiology holds good, namely, that the movement which results from external sensation is proportionate in extent to the force of the sensation, provided there be no natural hinderance, and the condition be that of health. But movements may follow on external impressions as simply nerve-actions, and which impressions are not objects of consciousness. But the law of proportional reaction and action holds good in these impressions also, as well as in sensations; the force, however, of the impression must not be estimated by physical laws, for a slight touch of a nerve may be and often is a forcible external impression, and vice versâ.

But besides these psychical actions resulting from external sensations, there is a class incidental thereto, which must be taken into consideration. For example, we connect with an external sensation the conception of another resembling it, which we have experienced before, and so there is an idea or image (Einbildung) in immediate connexion with the former, that commingles its psychical action in the mechanical machines with that proceeding directly from the external sensation. Thus we sigh if we see a person resembling another with whom we have experienced sorrowful feeling; the sigh is the psychical action of an "Einbildung," and only mediately of an external sensation. Further, we connect expectations or "Erwartungen" with our external sensations—expectations of others formerly connected with them, and there arises an anticipation (Vorhersehung) which commingles its influence in the mechanical machines with those connected immediately with external sensations. A certain person is accustomed to faint when bled; meeting the surgeon in the street he faints again. This faintness Unzer describes as a psychical action resulting from a "Vorhersehung" of a venesection, and only incidental to the external sensation of the surgeon.

Since all our external sensations are accompanied with pleasure or pain, and are associated with "Einbildungen," or inscribed ideas, and "Erwartungen," or expectations, so they consort with desires and aversions, and the psychical influence of these operate on the mechanical machines conjointly with that proceeding directly from external sensations. Thus the sight of food excites the sensation of hunger in a fasting person; peculiar tones or odours excite the sexual instinct, &c. On the other hand, strong sensational conceptions excite imperfect external sensations. Thus when we see a figure in the dark and take it for a ghost, and by this imperfect external sensation are affected with terror, and attempt to grasp it or repel it, the arm becomes spasmodically fixed, not directly by the true external sensation of the shadow or form, but incidentally by the imperfect external sensation caused by the appearance of the thing we take to be a ghost. If we reflect upon our external sensations, then conclusions of the understanding commingle in action with the former.

We pass over very reluctantly the physiology of mental action in its healthy and morbid states as detailed by Unzer. We can only remark that his explanations of the phenomena of dreaming, insanity, somnambulism, &c., and of the excitement of functional action by conceptions of the mind, whether of imagination, memory, anticipation, reasoning, &c., are quite as ingenious and plausible as any we have read, and what is curious we find that his illustrations are almost identical with those used by more modern neurologists.

*Action and nature of the desires and aversions.* A good deal of space is devoted to a consideration of the actions of the sensational desires and aversions through the nerves. The immediate psychical action of these is an effort of the cerebral power to produce the material idea of a foreseen sensation, if pleasurable, or its contrary, if painful; and it is the impression of pleasure or pain in the brain by the foreseen sensation which causes the effort. The psychical action of pleasant instincts and actions (joy) take place in harmony with, and in fitness or adaptation to, the animal machines; or if the impression be painful (sorrow), they are opposed to the natural function of the latter. Consequently, all pleasing instincts and passions are beneficial, or contribute to healthy action, and the painful the contrary. This proposition is of fundamental importance in understanding Unzer's views as to the instincts, propensities, and passions. Without defining pleasure or pain, he steadily adheres to the doctrine that those ideas and acts are pleasurable, which arise from impressions having a beneficial influence or beneficial results; and on the contrary, those ideas and actions are painful which are induced by impressions injurious to the organism, and opposed to the natural and healthy function of the part affected. Subsequently, he shows that the *feeling* of pleasure or pain is not requisite for the necessary actions in many animals, but that the law still holds good as to their excitement by the two kinds of external impressions, namely, the beneficial and the injurious. In illustrating the mechanism in the excitement of the instincts, he observes, that instinct for food, the satisfaction of which is the taking of food, saliva is poured forth: in the instinct to suckle, the satisfaction or contentment of which is the pouring out of milk through the nipple, by the act of sucking, there is an efflux of milk and an erection of the nipple. In infants the instinct to suck is exhibited in the action of the lips, which suck even at the air. So also ferocious animals, on the excitement of their instinct (rage), prepare their weapons of offence; in terror, the contentment of which is the escape from a great impending danger, the efforts are in accordance.

*Nature and operation of the sensational instincts.* Unzer's arrangement of the instincts is this: 1. The instinct of self-preservation which, consisting of strong desires, arising out of obscure (dunkeln) sensational impulses, and the object of which is the preservation (Erhaltung) and well-being of the animal. 2. Self-defence, which consists of strong aversions arising out of obscure sensational impulses, and the object of which is to avert death, or remove an unfavorable condition. 3. The propagation of the species, consisting of strong desires, and having the object of propagation of the species by union of the sexes. 4. The parental instinct, consisting of strong desires and aversions, and having for its object the maintenance, well-being, and defence of the offspring. In considering these in detail, Unzer classes the first two together, and breaks them up into minor or secondary instincts, such as love of life, instinct for food, or of hunger, thirst;—of dislike for certain food, or nausea; the instinct of motion of voluntary muscles to perform specific muscular acts, as singing, whining, leaping, &c.: the instinct for rest, the war-instinct (Wehr-trieb), instinct of destruction. We subjoin an account of the instinct of motion of voluntary muscles, as being novel, and also as illustrating the mode in which Unzer treats the subject.

"Nature has constituted bodily exercise of animals, a means of their maintenance and well-being; consequently, they are thereby in the healthiest state. If, therefore, to their disadvantage they neglect bodily exercise, a number of unpleasant external sensations arise, which we term indisposition, or sickness, and which all animals detest. This is the sensational stimulus of the *instinct of bodily exercise*, a stimulus which causes the vital actions to be more or less morbid according to its intensity, the pulse becoming feverish, and the breathing embarrassed. By this unpleasant sensational stimulus (which too much repose, or sleep, or nourishing diet, and many other causes may occasion) is the animal motived to strive for the opposite agreeable sensations, which, as it foresees (vorhersieht), must be attained by the movement of its muscles and limbs, and in this instinct, this is the object and peculiar design of nature, although it is ignorant of it [the object], and knows not for what ulterior purpose it must move its limbs. The psychical action of this sensational stimulus, insomuch as they constitute a foreseeing (Vorhersiehung) of bodily exercise, are displayed in the muscles of voluntary motion, that is, in those which an inner sensational impression from conceptions can move without the concurrence of another sensational impression being necessary; thereby they stimulate the same muscles to the movements which they accomplished perfectly during bodily exercise, and in the striving of the psychical forces after these same imperfect movements consist the psychical actions of the instinct to bodily exercise itself. This instinct to bodily exercise operates therefore in the machines which it formerly affected, namely, the muscles of voluntary motion of the limbs, whilst it impels the same to the natural actions of which they are specially capable, and which in particular, produces fully in them the satisfaction of the instinct, namely, bodily exercise. Consequently, in this instinct it is seen that the muscles jerk the limb, and the animal often seeks to hop, spring, soar, raise itself up, &c., in such manner as may happen perfectly by actual contentment of the impulse to bodily exercise, and if no extraneous hinderance occurs, commonly happens instantaneously, while the conception is only sufficient to move the voluntary muscles. In such cases, there follows through the connexion of physical, mechanical, and animal forces of the muscles set apart for voluntary motion, the further actions of the animal economy, namely, the changes in the composition of the fluids, the promotion of the secretions, the increase of muscular power, and of the sensational forces themselves, so far as they are in accordance with the objects of nature, which are treated of in detail in the physiology of the proper mechanism of animal bodies. (See Haller's Physiology, Second Discourse.)" (p. 268-70.)

Taking these views as a basis, Unzer proceeds to explain the various movements of animals, as the singing of birds, the cries of animals, laughter in man, the periodical migration of animals, the cleaning, pluming, &c. of birds and quadrupeds, the domestic architecture, the laws and practices of republican animals, &c. All these are allied to the instinct of voluntary movement, have certain obscure pleasing or displeasing external sensations and beneficial or injurious impressions, as a basis, by which the animal is motived to perform certain movements without knowing their object, but which, according to the plans of nature, through the assistance of external impressions, are really in the position to accomplish adaptively the maintenance and well-being of the animal. After discussing the respiratory instinct and showing its double character as a voluntary act, and a reflex act or nerve-action, Unzer discusses the instinct to repose and stimulation (zur Ruhe und Ermunterung); yawning, extending, stretching, are instinctive movements belonging to the latter, and not to the former. He then reviews other instincts, as the instinct of attack and defence (Wehrtrieb), of reproduction, of suckling—a branch of alimentative instinct, &c.; and next shows that there are general instincts, as the love of life, of pleasure, of food, of the propagation of the species; and special instincts proper only to certain animals, as respiration, care of the young, brooding, &c. Instincts may be unnatural or perverted, as self-destruction, sodomy, &c.; they may be also general or principal, and give the character to an animal, or a race of animals. The principal instinct is that which is most necessary for the well-being of the animal, as the Wehrtrieb or destructive instinct in carnivorous animals.

*Nature and action of the emotions and passions.* The affections, or passions, are analysed by Unzer, and he shows, that they differ from the instincts in this, that in the latter the animal is not necessarily conscious of sensorial stimuli, whereas in the former it is. Every animal is by nature impelled by a love of life, without knowing why. Nature has given them the means of protecting and maintaining their life without their knowing it, or its object, and they use those means unconsciously; but when they become conscious of the danger of death, they then experience the emotion termed fear of death (Furcht des Todes), and through this, other conceptions, desires, impulses, and emotions, are excited, all which combine for the preservation of life; so the *appetite* for food is distinguished from the *instinct* for food, and gives rise to other passions, as voraciousness and rapaciousness. So the *instinct* of attack and defence gives rise to the *emotion* of anger or revenge; the sexual instinct thus gives origin to the passion of love, and this to other instincts and emotions as combativeness, jealousy, &c. An agreeable emotion is joy, and Unzer draws the distinction between joyousness, contentment, or satisfaction, and hope. Sorrow, or unhappiness, on the contrary, is the emotion or feeling consequent on a disagreeable passion, and comprises the painful or injurious emotions. The whole of these are gone through *seriatim*, and their action on the mechanical machines pointed out. Then the animal dynamics of the understanding and of the will are discussed, and Unzer makes a remarkable distinction between movements which are purely spontaneous (freywillig), and those which are simply volitional (willkürlich) or dependent on sensations as well as on the will. This distinction is requisite to understand some of his views, as for example those regarding the instinct to voluntary movement. "There are," he observes "innumerable sentient volitional (willkürlich) movements which are not spontaneous (*freywillig*);" meaning apparently, that the movements are excited *in* the muscles of volition but not *by* a volition. A concluding chapter is devoted to the communion (gemeinschaft) of the body and the soul. In this short chapter, he separates the principles already expounded in the previous chapters, arranging them consecutively and referring each to the paragraph where it may be found evolved. The whole is too abstract for our pages, but is a singular example of lucid, profound, and logical thought. He adopts a medium course between the Stahlians and the mechanical physicians and materialists.

II. LAWS OF REFLEX ACTION. The second part of the book is devoted to a consideration of the nerve-actions, *actiones nervorum*, or in other words, of reflex phenomena.

> "Animal bodies become capable of movements by means of the forces of the nerves, which can neither be explained by the physical and mechanical laws of motion, nor deduced from the laws of the cerebral power, but are developed by the vital spirits, supplied to the animal machines, according to their own peculiar laws. To these belong those simply animal motions excited by external impressions on the nerves before they cause a material external sensation in the brain, with which the muscular irritability of Haller must be classed; and also those excited either by inner impressions on the medulla of the animal machines which develop no mental conceptions, or by other stimuli independently of ideas." (p. 343.)

*Action of centric and peripheral impressions and of the vis nervorum.* The introduction to the first chapter is entitled, "Of the forces of the nerves, and of the nerve-actions in particular." There are only two forces which act on the animal and mechanical machines, namely, the two kinds of impressions on the nerves. The external impression supplies to the mind all its external sensations but on this condition, that it reaches the brain, and excites therein material external sensations: and the inner impression produces all its psychical actions in the body under the condition that it be excited by conceptions. But should these conditions fail,—should the external impression not be propagated to the brain, or at least excite no external sensations therein; and should an inner impression on the origin of the nerves in the brain be not originated by conceptions, but by some other cause, are we then to conclude, asks Unzer, that the two kinds of impressions develop no animal action in the body?

"If a nerve, accustomed to be excited to the production of certain animal movements in the body, by certain external impressions that are perceived, be cut off from all connexion with the brain—the seat of the mind,—as for example, either by being tied, divided, or even by the entire separation of the head from the body; nevertheless (as we are taught by indisputable facts) so long as traces of animal life are left in the separated portion, the same external impressions from the point of impression to the point of division are the stimuli of the same animal movements, and actually develop them, although in such cases the external impression is never propagated to the brain, but reaches only to the point of division [of the head from the body], and consequently, no material external sensation can be excited in the brain, nor be perceived by the mind. This is the first of the fundamental principles upon which all depends, that will be propounded in this division of the physiology of animal nature." (p. 346.)

Unzer then proceeds to mention in rapid detail the experiments, which show the action of the vis nervorum or nerve-forces, in developing this class of movements. He mentions the contraction of a separated muscle, of a portion of intestine, of the heart; the secretion of the glands and the movements of viscera excited by a stimulus when separated from the body; the sexual actions excited in decapitated crickets when the organs of generation are irritated; the full performance of the sexual act by decapitated butterflies on the application of a proper stimulus, so that ova are deposited; and the retraction of the foot by a decapitated frog when pinched or injured. For an abundance of such facts, Unzer refers to Haller, Zimmermann, and Geder. He then in like manner shows, that inner impressions made on the nerves in any part of their course as far as their origin in the brain, are propagated to their peripheral extremities, and there excite animal actions just the same as if they had been excited by conceptions. The Italics in the subjoined extract are the author's.

"From these two fundamental principles, is deduced the irrefragable truth in the physiology of animal nature, that the animal machines are not destined by nature solely for the propagation of external impressions to the brain, to excite there the material ideas of sensations; nor destined to receive all their inner impressions from conceptions only: *but that they possess in addition another faculty altogether distinct, and one destined by nature for the production of those animal movements in the body by means of external impressions, which may be propagated to the brain, and be perceived, or which may not;* movements which are produced by the cerebral power when the impressions arrive there and are perceived; and further; inner impressions which they [the animal machines, or nervous system] derive from a touch or a stimulus, but which excite no act of the mind (Vorstellung), induce the same animal movements in the body, that are produced by the cerebral power, when an inner impression, identically the same, excites an act of mind. These animal machines are endowed by the Creator with the special animal motive-forces of the impressions, in a mode to us unaccountable and mysterious, partly to put the cerebral power into action, partly through these to move the body.

In fact, the greater portion of animal movements are so very much dependent upon these, even when they are at the same time psychical actions, that *it (the vis nervorum) must be received as the most general and the first principle of life, and of the whole organism.*" (pp. 352-3.)

*Analogy of the brain, spinal cord, and sympathetic ganglia.* In pursuing his subject, Unzer lays down the fundamental principles of reflex action, and carefully distinguishes between a reflex action and a psychical action. He points out how the stimulus of conceptions (the internal impressions) acting on the terminating fibrils of the nerves in the brain, and producing mental acts, may be superseded by a mechanical stimulus operating on the same fibrils, and developing the same results. And contrarily, the external impression on the nerve or on its trunk may or may not excite conceptions, but in either case movements are developed which are either nerve-actions only, or these and mental actions conjointly. He also points out that an external impression may excite reflex acts on its way to the brain, and when arrived at the brain it may there excite changes and psychical actions result. The independence of the vis nervosa of the brain is demonstrated, and a remarkable feature in Unzer's views is, that he includes the sympathetic or organic system of nerves in his doctrine of reflex action, the ganglia being centres of action. We think it far from improbable that his doctrine may be hereafter fully established. It is worthy notice too, that he considers the actions of those animals which are endowed with a ganglionic system only as displaying these reflex acts only, and that the mental acts are peculiar to the higher orders.

"This is the case most probably with all those animals in which there is no brain, but only nerve-like fibrils, capable of receiving impressions; as for example, the polypes whose nerves are not united into a brain, but interwoven with many ganglia. In animals possessed of a brain, each external impression which is perceived, goes direct to the brain, and excites therein a material idea, and a conception. But when in the brain it is inverted, or reflected as it were [gleichsam reflektiret] and returns back as an internal impression of the conception to the same nerve-fibrils that move the limb, which this external impression has the power to govern through a psychical action of its sensation. At the moment of this reflection when the external impression is changed into an internal, the act of thought takes place, and thus the movement which the external impression excites in the mechanical machines becomes a psychical action. When the polype receives external impressions, they pass directly forwards as far as the first ganglion, and here are altogether reflected as in a brain, or if only partially reflected then they partly reach to other ganglia, and so may be reflected many times. It is enough that at these points (the ganglia) the external are changed into internal impressions, and are propagated from the ganglia outwards to the mechanical machines which they set in motion without it happening, that during this change, a thought is excited, since there is *no brain*, for in that *only* a conceptive faculty is seated; and without the inner impression becoming a mental action, and operating as such. In such a manner, polypi may be able to perform all their animal movements, simply by external impressions on their nerves without consciousness on their part, or thought, and without either brain or mind." (pp. 360-1.)

We think this as explicit an account of the reflex acts of lower animals, as has ever been given. The existence of a special class of nerves may or may not be proved anatomically, but here it is shown that their anatomical distinctness is unnecessary. *The anatomical and physiological distinctness is claimed for the nervous matter in which they terminate.* In succeeding paragraphs Unzer propounds several modes in which mental actions may be changed into reflex actions. The retention of the urine is an act with sensation, but if the impressions exciting it do not arrive at the brain, they still act so as to close the sphincter. Thus the external impressions of worms in the stomach are not perceived; nevertheless, they pass to the central axis, become inner impressions, are reflected upon the motor nerves of the limbs, and excite convulsions (as in epilepsy) without any act of consciousness. But just the same epileptic convulsions may be excited by fear, &c.

In his answer to his Göttingen reviewer, Unzer brings forward a remarkable extension of his views. The reviewer, criticising Unzer's theory of the origin of corporeal or material ideas, observes—

"Corporeal ideas are developed at the origin of each nerve in the brain. It is, however, worthy notice that nerves arise also from the spinal cord and its gray matter, consequently their external impressions ought to be developed in it, yet it is quite certain that the soul is not seated in the cord, nor perceives by it."

Unzer's answer is very explicit.

"It is not a change only in the origin of a nerve excited by impressions which develops sensation and thought, but the cerebral structure is ever necessary to which the nerves must reach, if a sensation is to arise. Since new twigs frequently proceed from the ganglia, all may be sensible; but if that particular kind be wanting, which develops ideas in the brain, then the change which the nerves experience in the ganglia from external stimuli *is only a moving power* (Bewegendekraft), *a reflection of impressions upon other fibrils* which are regularly determined to action. *Just so is it with the nerves which arise in the spinal cord and have there probably the double function partly to transmit their impressions to the brain that sensations may be excited, and partly to be reflected on other nerves, so that thereby certain movements are developed which otherwise would not result from these impressions.*" (Answer to Review, p. 24.)

Nor is he less explicit as to the reflex function of the sympathetic ganglia. The reviewer observing that Unzer considered the ganglia to be small brains, Unzer explains in what sense he meant this. He ascribed to the brain various functions; in one only of these do the ganglia resemble it, namely, in having a reflex function.

"When an impression is developed in a nerve from without, it goes on to the brain, and is there felt. But this impression is forthwith reflected and is continued outwards along the nerve to the muscles. Consequently the perceived impression excites movements which are necessarily accompanied by sensation, or belong to the mind. The ganglia can in like manner reflect upon other nerves the external impressions which are propagated to them, and thereby move certain muscles. Now it is in this that they have a function similar to that of the brain, although during this reflex action no sensation is felt in the ganglia, as is the case during the reflex action of the brain. This is my real meaning." (Answer to Review, p. 59.)

Unzer devotes some space to a consideration of the question, whether the brain is endowed with the vis nervosa, or nerve-force, and whether it can give rise to nerve-actions, or reflex acts, without consciousness. He decides in the affirmative from several arguments: the most conclusive is founded on the fact, that injuries of the brain may take place without exciting sensation, and yet develop reflex acts, for a certain individual suffered from an exostosis, which penetrated into the brain; for some time there were no morbid phenomena, but at last death supervened on convulsions. The convulsions were caused, he argues, by the irritation; yet the person was not conscious of the cause nor of pain. The seat of the vis nervosa in the brain is the cortical substance, with which, he observes, that of the spinal cord is continuous, and the fact of its existence there is a fundamental fact. These facts have now no weight whatever, but it is worthy of notice that the phenomena of etherization corroborate in many instances the views lately propounded as to the occurrence of reflex cerebral action without consciousness.

*Analysis of reflex phenomena.* In further considering the reflex acts or "nerve-actions" Unzer cursorily notices those of the sensitive nerves, evidently thinking that a central impression is reflected to the periphery, and then the return of this to the brain excites a conception or sensation. But he dwells little on the subject, for this reason, that in general it is impossible to say *when* animals feel. The reflex-motor acts are therefore the most instructive, because they are visible. Unzer commences with the consideration of muscular irritability, which he firmly maintains to be a "nerve-action," in opposition to Haller, principally on the ground that it is impossible to separate the minute motor nerve-fibrils from the muscular, and that whenever a muscle or a portion of one is irritated nerve-fibrils are necessarily irritated too. And, as it is well known that irritation of a nerve-fibril will and can excite muscular action, there is no need for the doctrines of a vis insita. He, however, takes up Haller's arguments one by one and combats them. The contraction of a muscle from stimulation is a *direct* nerve-action; cardiac and vascular action is of this kind. That from the irritation of a nerve an *indirect* or "reflected." The extension of the doctrines of homæsthetic action to cell-structures, will give a new phase to the discussions carried on from time to time regarding the *vis insita.*

Having disposed of the muscular fibre, Unzer proceeds to show the reflex acts of the heart, arteries, alimentary canal, diaphragm, glandular structures, sexual organs, and limbs; and with reference to the latter he refers to experiments on decapitated animals, as insects, reptiles, and birds, up to man. "A decapitated man struggles for a few moments to undo his hands from the cords, to raise himself up, to stamp with his feet; a dove whose head has been struck off in flight, still runs on until it comes in contact with an obstacle: a frog leaps forward without a head," &c. The action of stimuli on the central axis, as the spinal cord of a frog, is also considered, and lucidly explained; and he shows that *certain acts excited by stimuli, may in their turn excite other reflex acts.* If the spinal cord of a decapitated frog be irritated by a needle, certain muscular acts are excited; but, he adds, "these acts now become external impressions and excite other reflex acts, and the animal raises itself up, leaps, swims, &c. according to circumstances."

*The incident-excitor and reflex-motor nerves and their functions.* Unzer defines the property of the nerves to receive external impressions, and excite reflex acts, and he distinguishes it from ordinary sensation or feeling by terming it "the external feeling? (Gefühl) of the nerves." The word gefühl cannot be translated by the term *feeling,* as is manifest from the context; the word homæsthesis (as defined before) would be the better expression. He observes that if Whytt and others had carefully distinguished the two kinds, they would never have renewed the old error of the ancients, that the soul was diffused through the body. The soul is that which conceives and thinks and feels, but the nerves (apart from the brain) can neither feel nor conceive. In like manner, there is an "inner sentient impression without consciousness," or an inner homæsthetic impression, acting upon the nerves at their point of connexion with the brain onwards to the muscles; or acting upon any part of the trunk of the nerve in the same direction. Unzer points out the injurious pathological errors which have resulted from a want of proper knowledge as to the functions of the nerves and the nature of reflex actions. Convulsions, epilepsy, paralysis, and, in particular, all diseases of the nerves have been thought to arise from morbid action of the brain, and their cure has been sought by remedies which act upon the brain; whereas they very often arise from reflected external impressions, of which there is no perception in the mind, and which operate independently of the brain; consequently the cure of the diseases noted should be attempted by removing the causes of the reflex action. After a most lucid detail and explanation of the phenomena of reflex actions from external and internal impressions, as produced by the vis nervosa and its laws of action, Unzer says that, on a consideration of all the phenomena, it is impossible to explain them otherwise than by the theory, *that in each nervous trunk there are two classes of fibres, the function of the one being to transmit impressions* TO *the central axis, the other to propagate them* FROM *the central axis.* There are examples of limbs totally paralysed to the will, which, when the nerve going to the limb is irritated, become convulsed, and contrariwise, an impression made on the periphery is transmitted and reflected without consciousness, with the effect of exciting convulsive movements on the powerless limb. These two classes of nerves he subsequently distinguishes as *afferent* and *efferent* (ableitenden and aufleitenden).

*Centric reflex action.* After devoting a paragraph to an announcement of "*the great truth that these impressions act in animals not endowed with consciousness or mind, and are the stimuli to their instincts,*" Unzer proceeds to notice the various modes of action of the "inner sentient impression without consciousness," and the nerve-actions, or reflex acts, that result. Thus the spinal cord can receive these impressions, for if that of a decapitated frog be irritated with a needle, the animal leaps, provided the continuity of the cord and of the nerves be perfect; but if they be divided, or crushed, no nerve-action results. An inner or centric impression, caused by the reflexion of an external impression, is subject to the same law. Such an example of nerve-action occurs, he says, when an external impression, caused by the external homæsthetic irritation of pinching a decapitated frog's foot, excites it to leap. "The external sensorial impression in this case is propagated uninterruptedly from the point touched in the toe to the point of injury in the spinal cord, and from this is reflected, and returns again uninterruptedly, and so the same nerve-actions are developed, as if another primary *internal* impression without consciousness had excited the spinal cord." Farther, the internal or centric impressions without consciousness are shown to act in the same way as conceptions themselves upon the origin of the nerves, and through the nerves upon the mechanical machines. Examples of centric reflex action illustrate this law, derived from the actions of the muscular and visceral systems. Thus, the repression of hemorrhage from the nose, by the application of cold to the neck or other part of the surface, is a reflex action upon the arterial capillaries. The act of respiration in the animals endowed with consciousness is partly voluntary, partly excited; but in the newly-born, in whom the brain is imperfect, and the mental faculty not developed, it is altogether an excited act. Vomiting from injury of the brain is a centric excited act; the injury acting as an internal or centric impression. The action of the lungs, and specially of the bronchi, is an excited act, as also is erection of the penis, and the emission of semen in epilepsy.

*Centric diffusion of impressions.* External impressions may be diffused, so as to induce convulsive action in muscles in other parts of the body of a decapitated animal than that in which the impressions are made. The bodies of animals, he says, are so constructed, that the "external sentient impressions with conceptions" may in their course to the brain be reflected upon other nerves and nervous twigs, and this also occurs with "impressions without conceptions," and the same movements result with them as with the former.

*Analogy between instinctive, reflex, and emotional actions.* The impressions of pleasure and pain have their analogues also in homæsthetic impressions, and the actions resulting from the former result also from the latter. For example, a tickling or a pain is caused by an external impression, and certain motions follow the sensation; the same impression without consciousness will produce the same results. Thus a still-born child or a suffocated person may have the respiratory act and the action of the heart renewed by irritating the nostrils; the same irritation would, if perceived, have caused a convulsive action of the respiratory machinery. In the same way, the acts characteristic of the instincts and emotions may be (and often are) only excited acts, and take place independently of conceptions, being excited by mere impressions, internal or external; consequently the great principle of self-preservation is manifest in the reflex acts, or nerve-actions. He argues that the impressions on the stomach of the decapitated turtle will cause automatically the acts which characterize the instinct of hunger on desire for food, namely, a creeping about as if in search for food, and a peristaltic action of the intestinal canal, although the *sensation* of hunger is not felt. The acts characteristic of the instinct to movement of the voluntary muscles, (see *ante*, p. 198) may also take place automatically, when the same impressions are applied which develop them normally. A living acephalous abortion will draw up its legs when they are pricked, and he says it is related that when a decapitated man was thrust through the heart by a sword, the arms were violently brought together; further, as soon as the headless trunk of a criminal falls to the ground it tries to undo its hands and struggles to get upon its feet; and that the same thing may be seen in a decapitated animal every day. He adds other examples, and says that they might be multiplied to any extent, remarking, however, that many of this class of movements, as the movements of sighing, crying, shouting, &c., are rendered impossible by decapitation, the "machines" suitable thereto being removed with the head.

In this manner Unzer passes in review the whole of the instincts, passions, and emotions, showing that the same law is in operation with all. With regard to the emotions and feelings, he observes, however, that only those acts can be excited by homæsthetic impressions which are excited by external sensations. The material ideas dependent on the operation of the conceiving force cannot be produced, that force being wanting, nor consequently can the movements corresponding to those ideas be excited. Thus, the acts characteristic of self-defence and of rage may be excited, and the weapons put into action automatically, by means of external impressions only.

Having considered what reflex act or nerve-actions replace voluntary actions, Unzer next shows that the latter may take the place of the former. He remarks that there are three principal kinds of reflex acts or nerve-actions: the first, are those arising from primary internal homæsthetic impressions; the second, from unperceived impressions reflected outwards; the third, from those impressions applied directly. He gives examples of these different kinds: when the spinal cord of a frog is irritated, movements are produced which in the natural state are psychical actions; this is an example of the first kind. Of the second kind is the leaping of a decapitated frog, when its foot is pricked. In discussing the mode in which this reflex impression operates, Unzer takes occasion to point out analogous acts. A man dreams that he is swimming, and acts in his muscles as if he felt that he was actually in water. A decapitated frog thrown into water will do the same thing. The effort of the man to swim is the imperfect psychical action of an imagined sensation (eingebildeten Empfindung), and may be an indirect nerve-action or reflex act of the external impression. So if a man dream he is touching burning iron he withdraws his hand from it, or if a youth experiences a venereal dream, the phenomena of the sexual instinct result from an external impression, just as decapitated animals will perform the same instinctive acts from a similar external impression. Examples of the third kind are muscular contractions, &c. on the direct application of stimuli, or when an intestine or other hollow muscle is stimulated to contraction. This part is closed with a chapter on "the communion or mutual action (Gemeinschaft) of the cerebral forces with the nerve-forces in the natural condition." It concludes with the following aphorisms:

"They err who infer that, because an animal performs animal-adapted actions (Handlungen), it must necessarily possess a soul, or the conceiving force, or volition, since it is undeniably possible that the nerve-forces (vis nervosa) alone can produce the greater number of animal-adapted actions. In consequence of this error, most philosophers have hitherto considered that all animals, without exception, have souls; nevertheless, according to all probability, many of them neither feel nor are conscious.

"It is not correct to infer, that those animal movements of animals endowed with thought and consciousness, which accompany their sensational conceptions, incitements, desires, aversions, &c., are psychical actions of the same, and cannot be nerve-actions; or vice versâ, because they are nerve-actions, they cannot be the psychical actions of these sensational conceptions. From this error, taken in connexion with the second mentioned in the previous section, the old error has probably arisen, which the author of the article 'Sensibilité,' in the Dictionnaire Encyclopédique, has revived, that there must be two souls in animals endowed with consciousness, namely, a rational, by which the psychical actions are produced according to physiological laws, and a sensational, from which the psychical actions of external sensations and other sensational conceptions proceed, and which are produced according to the laws of the nerve-forces. Nevertheless, a soul is requisite for the production of that which the nerve-forces effect.

"Lastly, those also err who conclude that, because an animal performs animal-adapted actions which are effected by the cerebral power alone, without the co-operation of the nerve-forces, that all animal movements are the actions of the psychical force. This is the error of the Stahlians, who maintain that all animal movements are psychical movements, nay, the adapted actions of a will, of which the soul itself is unconscious. On this same erroneous proposition depends the old error newly revived by Whytt, that the souls of animals must be diffused through the whole body along the nerves, because in decapitated animals the same animal movements can be produced, which previously were considered to be psychical actions," &c. (pp. 605-6.)

The THIRD PART of the work is devoted to a consideration of Animal Nature as a Whole. Unzer first defines the difference between vegetables and animals: he then divides animals into two principal classes, the one comprising those with souls, the other those without. The latter must be endowed with nerves, or with other machines having similar functions, and with nerve-forces, but not possessed of a brain, sensation, or consciousness. The animals with souls are divided into two classes, the one comprising the reasoning, the other the unreasoning or simply sensational animals. Both are endowed with nerves, vis nervosa, a brain and animal soul-forces, but the soul of the former is a spirit. The faculties of these various classes are then summarily stated in accordance with the principles demonstrated in the previous parts of the work.

In a second chapter, the principal genera of animals are marked out. Referring to the structure in certain insecta, arachnida, &c., which resembles a brain, he observes, that according to all probability it possesses only the vis nervosa, and *being analogous to the spinal cord and ganglia, like them reflects external impressions, receives internal without conceptions,* and by means of both, puts in motion the animal machines. The light, sonnds, savours, &c., which in animals endowed with sensations, excite a thousand conceptions and voluntary motions, excite in these animals the vis nervosa directly without the intervention of sensation, and the animal performs automatically the actions proper to various instincts. This position he defends through several pages, and explains the actions of republican insects, as ants, bees, &c., in accordance with the doctrine.

The third chapter is on the origin of animals, and is in fact a discussion of the phenomena of reproduction. The fourth is on animal life, and traces the development, growth, perfection, or period of puberty, and decline of the body. The fifth is on the system of forces of animal life, and is devoted to a consideration of the co-ordination and subordination of the various "machines" which they set in motion, the doctrines being in accordance with what has been previously laid down. Finally, the sixth and last chapter investigates the phenomena of death, or the cessation of action in the organism.

III. In this analysis, we have endeavoured to do justice to our author, but the reader will readily comprehend that the tone, spirit, and meaning of a book so comprehensive in its scope, and so profound in its principles cannot be fairly given in the space we have allotted to it. We can only say, that it has rarely been our good fortune to read and learn with so much pleasure and profit as on this occasion.

Perhaps a critical estimate of the merits of Unzer's system would properly close our analysis, and especially a comparison of it with those of more modern date. Such of our readers as are conversant with neurology, will have no difficulty in perceiving that Unzer was the teacher of Prochaska. They will also perceive that the doctrines of reflex action are clearly and lucidly propounded under another designation; that views brought forward in recent times as new are here perfectly expressed; and that if the doctrines advanced were adapted to the later discoveries in the anatomy of the nervous system, and the histological structures of the vital agents, they would constitute a more perfect system of vital dynamics than now exists. We will not, however, extend our remarks on this point, preferring to subjoin a biographical notice of Unzer from the pen of the distinguished Professor of Göttingen, Dr. K. F. H. Marx. We may add that this notice is the more valuable, and ourselves and readers are the more indebted to Professor Marx, inasmuch as no memoir of Unzer has been before published. A critical analysis of his works would be a curious addition to psychology, as it is evident that the truths he advanced when his judgment was mature, were only attained to after long and patient study.

John Augustus Unzer (or Untzer) was born April 29th, 1727, at Halle. In his thirteenth year, he exchanged school for the university of his native town, for the purpose of being educated as a physician. He attached himself principally to Professor Funker, who initiated him into the Stahlian doctrines and taught him at the bedside. Unzer himself studied more especially the works of Boerhaave.

When only eighteen, and yet a student, he tried his hand at authorship, and published anonymously, 'Neue Lehre von der Gemuths Bewegungen, Halle, 1746' (New Views regarding Mental Operations), in which he attributed the emotions to varying tension of the nerves. In the same year he wrote a defence of the theory of Stahl, entitled 'Gedanken vom Einflusse der Seele in ihren Körper,' 1746 (Thoughts on the Influence of the Soul on the Body). He also published 'Gedanken vom Schicksale der Gelehrter' (Thoughts on the Fate of Scholars), and also 'Gedanke vom Schlafe und den Traümer nebst einem Send-schreibe, dass man ohne Kopf empfinden könne' (Thoughts on Sleep and Dreams, together with an Essay, showing that there may be sensation without the head), under the somewhat mysterious signature of "S. C. I. S." [Sine capite inducitur Sensatio?] The year following (1747), he published 'Abhandlung vom Seufzen' (A Treatise on Sighing.) On taking his doctor's degree, 9th Sept. 1748, he defended his dissertation 'de Sternutatione,' and the following year the dissertation 'de Nexu Metaphysices cum Medicina generatim.' In 1750, he published 'Philosophische Betrachtung des menschlichen Körpers überhaupt' (A Philosophical Consideration of the Human Body generally.) Towards the close of this year, he left Halle, and went to Hamburgh, where he immediately became connected with the 'Hamburgischen Magazin' (vide the 6th volume), but as he soon got into practice in the adjoining town of Altona, he resided there.

In 1757, he married Johanna Charlotte Ziegler, a poetess, who had the laurel crown conferred on her at Helmstadt in 1753, and who was also a philosopher. For the purpose of obtaining a proper estimate of physicians and medicine, of extending sound medical knowledge, of removing prejudices, and of checking the practices of ignorant practitioners, Unzer resolved to establish a weekly periodical, entitled 'Der Artz, ein Medicinische Wochenschrift.' Being edited in an excellent spirit, and with singular talent in the style and getting up, it exercised a beneficial influence in foreign countries as well as in Germany, for it was translated into Danish, Swedish, and Dutch. It appeared during the years 1759-64 in 12 parts, and to the first a portrait of Unzer, by Tuchbein, was appended.

In 1766, he published 'Sammlung kleiner physicalischer Schriften,' (the third appeared in 1769); in 1768, 'Grundriss einer Lehrgebäudes von der Sinnlichkeit des thierischen Körper;' in 1771, appeared his 'Erste Gründe einer Physiolgie der eigentlicher thierischen Natur thierischen Körper.' This work is probably his most distinguished performance. It is worked throughout in a philosophic spirit; is clear, precise, profound. He simply endeavoured to elucidate without too many general assumptions the obscure doctrines regarding the nervous system, particularly of the ganglia, and the *reflex actions.* He carefully modified his early unconditional assent to the views of Stahl, and specially sought to explain the stimulation of the nerves by natural laws. The animal movements are not mental actions.

As Unzer was in extraordinary request as a physician, on account of his skill, and as a man, on account of his agreeable sociableness, in 1772 he called Dr. I. C. Unzer, his nephew, to his assistance, who died in 1808 at Göttingen, on an excursion to the baths.

In 1773, Unzer edited 'Physiologische Untersuchungen auf Veranlassung der Recensionen seiner Physiologie.' In 1778, appeared 'Ueber die Anstechung, besonders der Pocken, in einer Beurtheilung der Neueren Hoffmannischen Pocken-theorie,' (this was translated into French, in extracts in Vickler's Mémoires sur les Maladies contagieuses. Strasbourg, 1786.) C. H. Hoffman considered certain glands of the skin to be pock-glands, whose function was to secrete a fluid, which by putrefying generated the contagion of smallpox. On the 29th January, 1782, his wife died in the 58th year of her age. In the same year, he published 'Einleitung zur allgemeine Pathologie der anstechenden Krankheiten,' (a copious and substantial work.) In 1783, appeared 'Vertheidigung seiner Einwürfe gegen die Pockentheorie von Hoffmann.' He died, a rich man, aged 72 years, on the 2d of April, 1799.

